# Patents and Patentees

**Published:** 1853-01

**Authors:** J. W. Keyes

**Affiliations:** Quincy, Florida.


### ARTICLE XII.

*Patents and Patentees.* By J. W. KEYES, M. D., D. D. S., of Quincy, Florida.

WE hold that no one has a *right* to do any thing which may, in any wise, detract from the dignity and respectability of the family to which he is attached, the circle in which he moves, or the community in which he lives. That any innovation upon the established rules which govern these associations, can only properly be made by and with the consent of the parties, and any otherwise made should subject the innovator to excommunication, unless he repent and make reparation.

Next to the minister of God, in dignity, and purity, and honor, and all the high and holy graces, should stand the enlightened and conscientious member of the healing art. For who, next to the minister, can approach so nearly perfection in all that adorns and beautifies human nature, "as he who, full of charity and love, goes forth with the exalted hope, the high resolve to do what he can to alleviate the suffering of his fellow." In what other profession or capacity can a man show himself so clearly above the base and sordid things of earth, in what so extendedly exercise that charity, which so elevates and adorns our nature? In what other vocation should the noblest impulses of his nature be so constantly called into action ? Is it little else than sacrilege to pervert this high calling into a merely money making means ? To shut out from the heart the noble feelings and cultivate only those which tend to debase, repress the love of doing good and cultivate the love of money and of self?

These thoughts have been suggested by reading Leslie's "Report" of the proceedings of the Mississippi Valley Association of Dental Surgeons, and Kendall on "American Dental Patents," published in the Journal. We cannot undertake to decide whether Dr. Leslie has correctly reported the M. V. A. D. S. or *not*, but we may and do declare that the principles for which he contends are those which should govern our profession.

A physician who would patent any medicine, compound or instrument which he believes will be of great importance in the alleviation of disease and suffering, should be regarded in the same light, held up to the same scorn and contempt as a minister of God who would patent a way of salvation.

In the dark ages of our profession, patents, nostrums and secrets could be winked at, but now, in this enlightened era, there is *no* excuse, and he who resorts to such a course to obtain notoriety and gain, merits the opprobrium and obloquy of his brethren, and should have the hand of fellowship withheld, should be regarded as a renegade and treated as an outlaw. As we have said, in years gone by, the sin of a patent was not so apparent, but now, that infant dentistry has grown to years of discretion and is a child not unworthy the noble mother from whom she sprung, her devotees should not disgrace her by befouling themselves with things unclean or habits unholy. And henceforth, any member of the profession who shall be so lost to a just sense of right, so forgetful of his high calling, so regardless of what is due to himself and to mankind, as to patent any improvements or hold any secrets in the art of healing, should be held at the same distance, regarded with the same disgust and loathing as that horde of contemptible soulless creatures, who, by means of their patent nostrums, thrust, filchingly thrust, their polluted lucre-loving hands into the pockets of the poor, the honest, the ignorant, the sick and dying. The great and growing evil, patent remedies, which is so blightingly cursing our land, could never have grown to its present extent but for those honorless members of the medical profession, who, by their example and countenance, have given it credit among the people.

Thus will the dental profession be cursed with an emanation, a fungus potent in evil, unless its members set their faces as a flint against it. It is with much pain and sorrow, that in looking over the list of *dental* patents, we find the names of some, coupled with patents whose talents and attainments entitle them to a high, useful and ornamental place in the profession. How different the feelings which arise in contemplating the character of the man, who, having devoted time, talent and money to the improvement of some branch of practice freely *gives* it to his brethren and the world. Who, contrasting the professional career of Hullihen or Taylor with Allen or Hill, does not feel his heart warm towards the genuine and philanthropic devotee to general good, and chilled towards the self loving, gain getting patentee?

The day has arrived when those who love their profession, and would lend their aid to elevate and improve it, should eschew the patent as they would dishonesty; should hold themselves aloof from every species of quackery, trusting wholly to moral and professional merit for success.

We would apply the proposition with which we set out, and maintain, that a dentist has no *right* to patent any improvement. And we say he has no *right*, because it is a violation of the ethics of his profession, and because he owes to the profession the best energies of his mind and the benefit of his experience. He owes, justly owes, this as a return for all the knowledge which he has received from the profession. Where, we ask, would we be, if all who have preceded us had hoarded and secreted what they know! If all had been actuated by the same spirit which actuates the patentee, how far would the *science* of dentistry be advanced?

Suppose those who have so nobly devoted themselves to the elevation of the profession, had been governed by the same sordid desires, the same insatiable thirst for gain, how far in dignity, usefulness and respectability would dentistry be advanced? How many journals would be supported, how many schools established?

Think of it, sirs, would not the course which you pursue have kept the profession where it was thirty or forty years ago?

---

### SELECTED ARTICLES.

